# Placental Underperfusion in a Rat Model of Intrauterine Growth Restriction Induced by a Reduced Plasma Volume Expansion

**DOI:** 10.1371/journal.pone.0145982

**Published:** 2016-01-04

**Authors:** Karine Bibeau, Benoit Sicotte, Mélanie Béland, Menakshi Bhat, Louis Gaboury, Réjean Couture, Jean St-Louis, Michèle Brochu

**Affiliations:** 1 Department of Molecular and Integrative Physiology, Université de Montréal, Montréal, Québec, Canada; 2 Department of Pathology and Cellular Biology, Université de Montréal, Montréal, Québec, Canada; 3 Department of Obstetrics-Gynecology, Université de Montréal, Montréal, Québec, Canada; University of Quebec at Trois-Rivieres, CANADA

## Abstract

Lower maternal plasma volume expansion was found in idiopathic intrauterine growth restriction (IUGR) but the link remains to be elucidated. An animal model of IUGR was developed by giving a low-sodium diet to rats over the last week of gestation. This treatment prevents full expansion of maternal circulating volume and the increase in uterine artery diameter, leading to reduced placental weight compared to normal gestation. We aimed to verify whether this is associated with reduced remodeling of uteroplacental circulation and placental hypoxia. Dams were divided into two groups: IUGR group and normal-fed controls. Blood velocity waveforms in the main uterine artery were obtained by Doppler sonography on days 14, 18 and 21 of pregnancy. On day 22 (term = 23 days), rats were sacrificed and placentas and uterine radial arteries were collected. Diameter and myogenic response of uterine arteries supplying placentas were determined while expression of hypoxia-modulated genes (HIF-1α, VEGFA and VEGFR2), apoptotic enzyme (Caspase -3 and -9) and glycogen cells clusters were measured in control and IUGR term-placentas. In the IUGR group, impaired blood velocity in the main uterine artery along with increased resistance index was observed without alteration in umbilical artery blood velocity. Radial uterine artery diameter was reduced while myogenic response was increased. IUGR placentas displayed increased expression of hypoxia markers without change in the caspases and increased glycogen cells in the junctional zone. The present data suggest that reduced placental and fetal growth in our IUGR model may be mediated, in part, through reduced maternal uteroplacental blood flow and increased placental hypoxia.

## Introduction

The aetiology of abnormal fetal growth, i.e. intrauterine growth restriction (IUGR), is not well understood, with about 40% of IUGR cases being idiopathic [1]. Remodeling of the uterine vascular bed and formation of placenta are important events in fetal growth and development. In the idiopathic IUGR, lower maternal plasma volume expansion has been found [2]. However, little is known about the impact of plasma volume expansion on remodeling of uterine arteries and placental development.

The glycogen cells (GC), a trophoblast subtype, are detected in junctional zone of the rat placenta at day 15 of pregnancy and the number declined from day 18, most of them disappear before parturition [3, 4]. GCs migrate to the decidua invading the spiral arteries to erode the muscular arterial walls [5]. Presence of these cells in term-placenta suggests abnormalities in placental development and possibly alteration of uterine vascular bed remodeling. Moreover, the number of clusters of glycogen cells or cytolysis of these cells was increased in placenta from pregnant rats exposed to toxicants [4].

The expansive remodeling undergoing during normal pregnancy leads to an increase in uterine blood vessel diameter [6, 7] and a reduction in blood flow resistance within this vascular bed [8]. Concomitant with these structural changes is an increased response to vasoconstrictors such as angiotensin II and phenylephrine [7, 9, 10]. Myogenic tone, an intrinsic property of vascular smooth muscle which contracts in response to stretching, is also increased in the radial artery of term pregnant rats compared to non-pregnant counterparts [9, 11]. This is thought to contribute to an adequate fetoplacental circulation [12] by establishing basal vascular tone and autoregulation of blood flow [13]. In humans, a low resistance uteroplacental circuit with a relatively elevated blood velocity in diastole has been shown [14–16] and can be evaluated by comparing diastolic and systolic blood velocity waveforms by ultrasound assessment of uterine circulation. Downstream resistance, derived from abnormal placental development and/or uterine vascular remodeling failure, results in a lowering of diastolic relative to systolic blood velocity [17]. Abnormal uterine artery flow in the first and second trimester have been associated with subsequent adverse pregnancy outcome, including preeclampsia and fetal growth restriction [15].

Clinical and basic research data suggest that IUGR results from an altered invasion of uterine spiral arteries, which increase uteroplacental vascular resistance and may compromise placental perfusion as well as maternal transport of nutrients and oxygen (O_2_) [18]. Low oxygen tension activates hypoxia inducible factor (HIF-1α) that, in turn, stimulates the expression of multiple genes including VEGF and its receptors [19]. In normal pregnancies at high altitude, increased HIF-1α mRNA and protein expression in placenta as well as increased maternal circulating VEGF concentration were associated with reduced birthweight [20]. Thus, HIF-1α and VEGF are markers of placental low oxygen tension. Apoptosis was enhanced by hypoxia in cultured trophoblast cells [21] and its incidence was increased in IUGR placentas compared with normal third-trimester placentas [22].

Late gestation rat cardiovascular adaptation and blood flow to uterine tissue are qualitatively similar to those occurring in the first trimester of human gestation [8]. Moreover, rat and human trophoblast-directed remodeling of uterine spiral arteries exhibits striking similarities qualitatively occurring in the last week of gestation in rats and in the first trimester in human [23]. In our laboratory, an animal model of IUGR was developed by administering a low-sodium diet to rats during the last week of gestation [24]. This model is well-established for the last 15 years and always showed reduced maternal circulatory volume accompanied by a decrease in uterine artery diameter and placental weight compared to normal pregnant rats [10, 24, 25]. Litter size is not affected and no fetal involution occurs with the interventional diet [24, 25]. Maternal food consumption is not different between the two groups [24, 25]. Therefore, this low-sodium diet was used as a tool to prevent plasma volume expansion normally occurring during gestation, making our model relevant to the human condition. Rat placentation is associated, as in human, with both interstitial and endovascular trophoblast invasion which is initiated at around embryonic day 13 and reaches a peak on gestational day 18 [3, 26]. In the rat IUGR model used by our group, maternal treatment is initiated on day 15. In light of the aforementioned observations, we hypothesized that, in the present IUGR model, the absence of blood volume expansion disturbs uterine radial artery remodeling and myogenic response leading to reduced uteroplacental perfusion. This in turn compromises nutrient supply and may cause hypoxia in the placenta. The first aim of the current study was thus to characterise uteroplacental perfusion by measuring blood velocity in the main uterine artery and to evaluate the diameter and myogenic function of the radial artery in our IUGR model. The second aim was to determine the presence of hypoxia, apoptosis and glycogen cells in control and IUGR term-placentas.

## Materials and Methods

### Animals and tissue preparation

This study was carried out in strict accordance with the recommendations of the Canadian Council on Animal Care. The protocol was approved by our institutional Animal Care Committee (CHU Sainte-Justine, permit number: R09-55). All efforts were made to minimize suffering. Female Sprague-Dawley rats (Charles River Canada, St-Constant, Quebec, Canada) weighing 225–250 g were mated with a known fertile male. Day 1 of pregnancy was determined by the presence of spermatozoa in morning vaginal smears. All animals were housed under controlled lighting (6 AM-6 PM) and temperature (21 ± 3°C). The dams were randomly assigned to 1 of 2 diets for the last 7 days of gestation (term = day 23). The control group was fed a normal diet containing 0.20% sodium and 0.40% potassium (normal diet 5755; PMI Feed Inc., Ren’s Feed and Supplies, Oakville, Ontario, Canada) and tap water. The second group, the IUGR group, received a low-sodium diet containing 0.03% sodium and 0.85% potassium (low-sodium diet 5881; PMI Feed Inc.) and demineralised water. The composition of both control and experimental diets was similar in protein (19%), carbohydrate (60.6%) and fat (10%) content. On day 22 of gestation, animals were sacrificed (8AM-9AM) by decapitation. The uteri were removed and placed in a dissection dish containing cold HEPES-buffered physiological salt solution (PSS) composed of (in mM): 130.0 NaCl, 4.0 KCl, 4.0 NaHCO_3_, 1.8 CaCl_2_, 1.2 MgSO_4_, 1.18 KH_2_PO_4_, 10.0 HEPES, 0.03 EDTA and 5.5 glucose (pH = 7.4). Placentas (n = 1 represents 3–4 placentas per dam) were quickly removed and snap-frozen in liquid nitrogen. For histology and immunohistochemistry, placentas were immediately immersed in 10% formalin and subsequently embedded in paraffin.

### Doppler recordings

Pregnant rats (n = 8 in each group) were imaged transcutaneously on gestational days 14, 18 and 21 using an ultrasound system (ACUSON CV70 system, Siemens Canada Limited, Montreal, Qc, Canada) and a 15-Mhz linear probe operating at 18–20 frames/s. In Doppler mode, pulsed repetition frequency was set at 13-Khz and a 0.5-to 0.9-mm Doppler gate was used. The pregnant rats were anesthetised with 2% isoflurane in 100% oxygen at 1L/min by face mask during ultrasound exams. Temperature was maintained with a heating pad. All hair was removed from the abdomen by shaving and conductive gel was used as a coupling medium. Studies were performed between 9AM to 11 AM and lasted less than 45 minutes for each animal. Pregnant rat main right uterine artery and umbilical artery from one fetus were visualized in colour mode, followed by blood velocity waveform recording with an angle of <42°. Peak systolic velocity (PSV) and end diastolic velocity (EDV) were measured and the resistance index (RI) was calculated (RI = (PSV-EDV)/PSV) on three cardiac cycles and averaged.

### Myogenic responses of the uterine radial artery

Segments of uterine radial arteries supplying a placenta were dissected from surrounding tissues under a stereo dissection microscope and transferred to the chamber of a small-vessel arteriograph (CH\2\M, Living System Instrumentation (LSI), Burlington, VT, USA). Both ends of the vessel were tied onto glass PSS-filled cannulas. The proximal cannula was connected to a pressure servo control unit (PS200, LSI). Residual blood was flushed from the lumen and the distal cannula was shut off with a stopcock. Temperature was maintained at 37°C with a temperature controller (TC-01, LSI). The chamber was positioned on an inverted microscope (Nikon eclipse TS100) and the internal diameter and apparent wall thickness of the arteries were measured by a video dimension analyser (V94, LSI) in conjunction with a data acquisition system (BIOPAC MP 100A-Ce System Inc, Santa Barbara, CA, USA).

A total of 18 radial artery segments from 9 rats in each group were studied. Arteries were equilibrated for 30–40 minutes without flow, with changes in PSS every 15 minutes. Pressure changes from 40 to 80 mmHg for 5 minutes were performed in order to verify the development of myogenic tone. Myogenic response was assessed by recording pressure-diameter curves. Luminal pressure was lowered to 10 mmHg and increased in incremental steps of 10 mmHg between 10 and 100 mmHg at 5 min intervals. The internal diameter was recorded at the end of each interval. After this procedure, to verify the integrity of the vessel and the endothelium, arteries were returned to 40mmHg pressure and contracted with phenylephrine (Phe, 1μM). When stable contraction was reached, carbachol (Cbc, 100 μM) was added to obtain a relaxation confirming presence of functional endothelium. Arteries that did not respond adequately to PE and Cbc were discarded. The pressure diameter curve was repeated in calcium-free PSS containing 2 mM EGTA (PSS EGTA). Percent myogenic tone (%MT) was calculated using the following formula at each pressure step: %MT = (D_1_-D_2_)/D_1_ X 100, where D_1_ is the internal diameter in PSS EGTA and D_2_ in presence of 1.8 mmol / L CaCl_2_ PSS. In order to determine an index of distensibility (ID), the fractional changes in lumen diameter as a product of pressure was calculated from lumen diameter measurements using the formula: ID = (Di − Do)/Do where Di is the internal diameter at a given pressure and Do is the diameter at 10mmHg.

All salts used in the above experiments were of analytical grade and obtained from Fisher Scientific (Montréal, QC, Canada). Phe (phenylephrine hydrochloride) and carbachol (carbamylcholine chloride) were purchased from Sigma Aldrich (Oakville, ON, Canada).

### Real-Time Quantitative PCR (qPCR)

Total RNA was isolated from frozen placental tissue using Qiazol reagent according to the manufacturer's instructions RNeasy Lipid Tissue Mini Kit (Qiagen, Toronto, ON, Canada). Integrity of the samples was ascertained by the A260/280 and A260/230 ratio.

PCR primers specific for genes of interest (Table 1) were designed with PRIMER3 (www.genome.wi.mit.edu/cgi-bin/primer/primer3_www.cgi) based on sequence data from the National Center for Biotechnology Information and obtained from Life Technologies (Burlington, ON, Canada).

**Table 1:** PCR primers designed for genes of interest.

Gene	Primer sequence
**Gene number**	
**House keeping gene (18S)**	FP: TCA ACT TTC GAT GGT AGT CGC CGT
**NM_X0117**	RP: TCC TTG GAT GTG GTA GCC GTT TCT
**Hypoxia inducible factor 1, alpha**	FP: TAG ACT TGG AAA TGC TGG CTC CCT
**subunit (Hif1α)**	RP: TGG CAGTGA CAG TGA TGG TAG GTT
**NM_024359**	
**Vascular endothelial growth**	FP: AGT GGC TAA GGG CAT GGA GTT CTT
**factor A (Vegfa)**	RP: GGG CCA AGC CAA AGT CAC AGA TTT
**NM_BC168708**	
**Kinase insert domain protein receptor (Kdr) (VEGF R2)**	FP: AGT GGC TAA GGG CAT GGA GTT CTT
**U93306**	RP: TTA CAC GTC TGC GGA TCT TGG ACA

FP, Forward primer (5’ → 3’); RP, Reverse primer (5’ → 3’)

Single-strand cDNA was synthesized according to the procedure in the QuantiTect Rev Transcription Kit (Qiagen Toronto, ON, Canada). Q-PCR reactions were carried out using the Biotool SYBR^®^ Green (Biotool, Cedarlane, Montreal, QC, Canada) and specific primers. The mRNA levels were normalized to 18S expression levels. The targeted and referenced genes were amplified in duplicate in the same run using the Mx3000P Q-PCR System (Stratagene, CA, USA). For each placenta, extraction and amplification were done three times. The relative quantification of target genes was determined using the MxProTM Q-PCR software version 3.00 (Stratagene). Briefly, cycle threshold (Ct) average of each duplicate was calculated for each gene and 18S and the ΔCt (Ctgene—Ct18S) was determined. The control placental tissue sample was chosen as a reference sample and set as 100% of gene quantity. The relative quantification of gene expression was analysed by the 2^- ΔΔCt^ method.

### Protein expression

Frozen placentas were homogenized in commercial lysis buffer (1:4 w/v) and centrifuged at 12,000 *g* for 15 min at 4°C. VEGF protein concentration was determined on supernatants by an ELISA commercial kit according to the procedure detailed by the manufacturer (RayBio^®^ Rat VEGF Elisa kit, RayBiotech, Inc., Norcross, GA).

### Histology

Formalin-fixed paraffin-embedded tissue sections (4μm) were cut and stained with hematoxylin and eosin for histological examination. Slides were digitized using the NanoZoomer 2.0-HT slide scanner (Hamamatsu Photonics, Boston, MA, USA) and analysed using the software NPDview2 (Hamamatsu Photonics). Number and area of clusters of glycogen cells were evaluated on two sections per placenta, and six placentas from different litters in each group.

### Immunohistochemistry (IHC)

IHC analyses on formalin-fixed paraffin-embedded tissue sections (4μm) were carried out using the automated DiscoveryXT staining platform from Ventana Medical Systems (Tucson, AZ, USA). Primary rabbit polyclonal Caspase-3 (cleaved) antibody was obtained from Biocare Medical (CP229; Concord, CA, USA) and used at a 1:200 dilution for 32min at room temperature (RT), after standard heat-induced epitope retrieval (HIER) with Cell Conditioning 1 solution (CC1). Primary rabbit polyclonal Caspase-9 antibody was purchased from Sigma Aldrich (HPA001473; Oakville, ON, Canada) and used at a 1:50 dilution for 6h at RT, after standard HIER with CC1. Biotin conjugated anti-rabbit secondary antibody from Jackson ImmunoResearch Labs (711-065-152; West Grove, PA, USA) was applied for 32min at RT and detected using the ChromoMap DAB detection kit (Ventana Medical Systems). Slides were counterstained with Hematoxylin for 4min and post counterstained with Bluing Reagent for 4min. Immunostained slides were digitized using the NanoZoomer 2.0-HT slide scanner (Hamamatsu photonics). Quantitative image analysis was performed with the Visiomorph DP software (Visiopharm, Broomfield, CO, USA). The junctional and labyrinth regions of each placenta were evaluated independently and defined manually as specific regions of interest (ROI). Detection of DAB signal was done using HDAB-DAB color deconvolution and intensity parameters adjusted to generate proportional scores (Negate +255 for 8-bit values). Within the ROI, an unsupervised k-means cluster analysis was performed to separate pixels into 4 classes corresponding to negative, low, moderate and strong IHC staining. Mean Intensity (MI) and Area were defined for each class and the global score (GS) for a given ROI was calculated as:

GS = (MI x Area)LOW + (MI x Area)MOD + (MI x Area)STRONG / Total Area of ROI. Six placentas from different litters were analysed for each group.

### Statistical analysis

Results are expressed as mean ± SEM. Results from Doppler and arteriograph studies were compared by two-way ANOVA for repeated measures followed by Bonferroni post-test, while the remaining data were compared by Student's *t* test or one sample *t* test when appropriate. Statistical analyses were performed using GraphPad Prism software version 4.03 for Windows (GraphPad Software, San Diego, CA, USA). Statistical significance was assumed with a value of *P*<0.05.

## Results

### Main uterine artery blood velocity in control and IUGR pregnant rats

**Fig 1:**
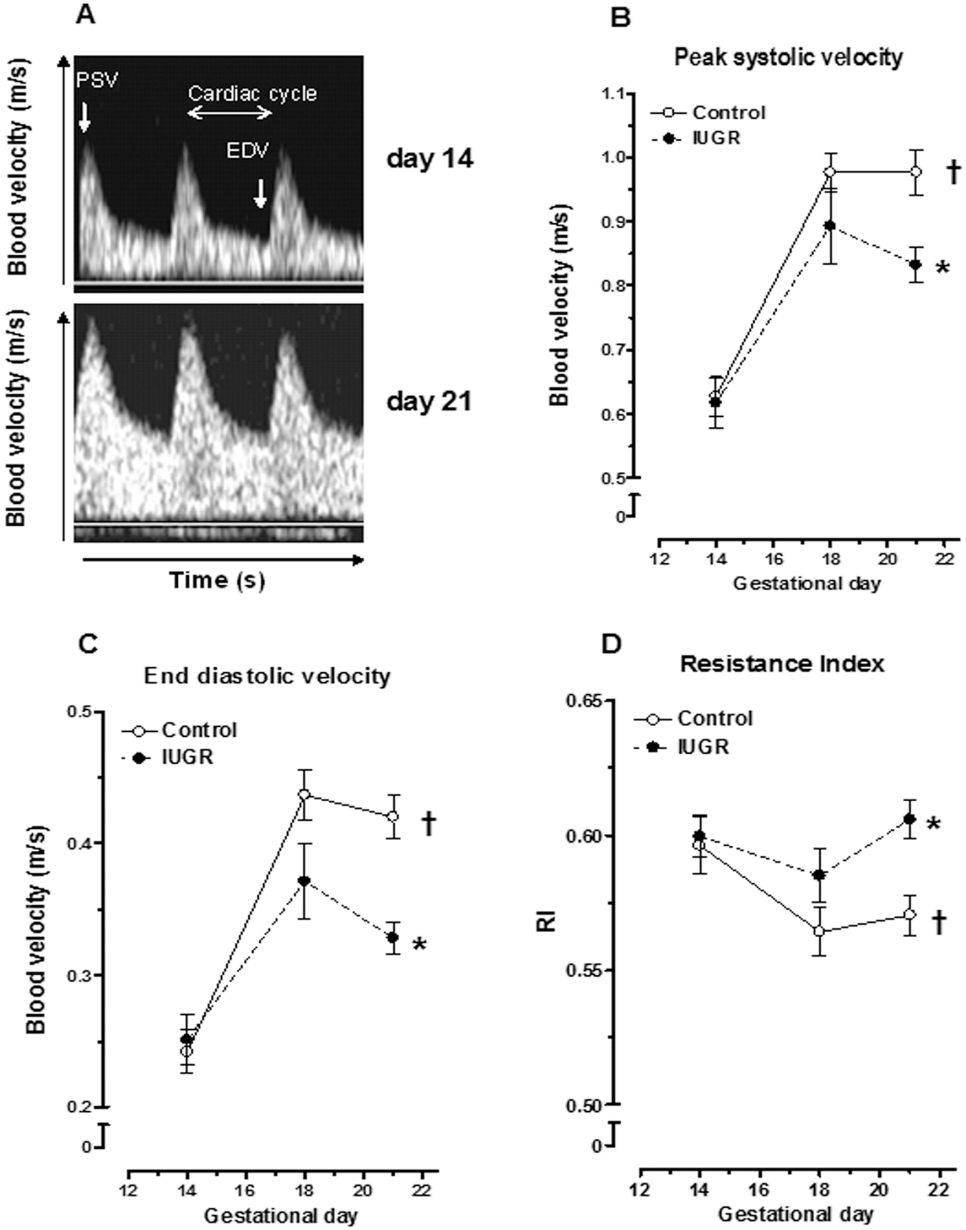
Uteroplacental circulation in control and IUGR pregnant rats. (A) Doppler flow velocity waveforms obtained in the uterine artery of a control rat at day 14 and 21 of gestation. (B) Peak systolic velocity, (C) end diastolic velocity and (D) resistance index in the uterine artery. Results are expressed as means ± SEM from 8 animals/group. † P<0.05, two-way ANOVA, effect of gestational day; * P<0.05 vs. control pregnant rat, Bonferroni post test. PSV: peak systolic velocity; EDV, end diastolic velocity; RI, resistance index.

To characterise uteroplacental perfusion, uterine artery blood velocity was assessed using Doppler ultrasound. At day 14 of pregnancy (Fig 1A, upper panel), the main uterine artery blood flow waveform was characterised by an abrupt increase in systole velocity followed by a progressive decrease ending in a relatively high end diastolic velocity (EDV). There were no changes in velocity waveform shape over the next seven days except for higher values along the cardiac cycle (Fig 1A, lower panel). Indeed, peak systolic (Fig 1B) and end-diastolic velocity (Fig 1C) in the uterine artery increased from day 14 to day 18 and stabilizing at day 21 (P<0.05, two-way ANOVA) during normal gestation. However, in the IUGR group, both peak velocity and EDV at day 21 of pregnancy were reduced in the main uterine artery (P<0.05, Bonferroni post-test). In normal pregnancy, as blood velocity increased, resistance index (Fig 1D) was simultaneously reduced (P<0.05, two-way ANOVA). In the IUGR group, resistance index values at day 21 were increased back to those observed in day 14 pregnant rats. This increase in impedance was statistically significant (P<0.05, Bonferroni post-test). Maternal heart rate did not differ between the two groups of pregnant rats (data not shown). The above results hence suggest a decreased uteroplacental perfusion in IUGR rats.

### Umbilical artery blood velocity in control and IUGR fetuses

**Fig 2:**
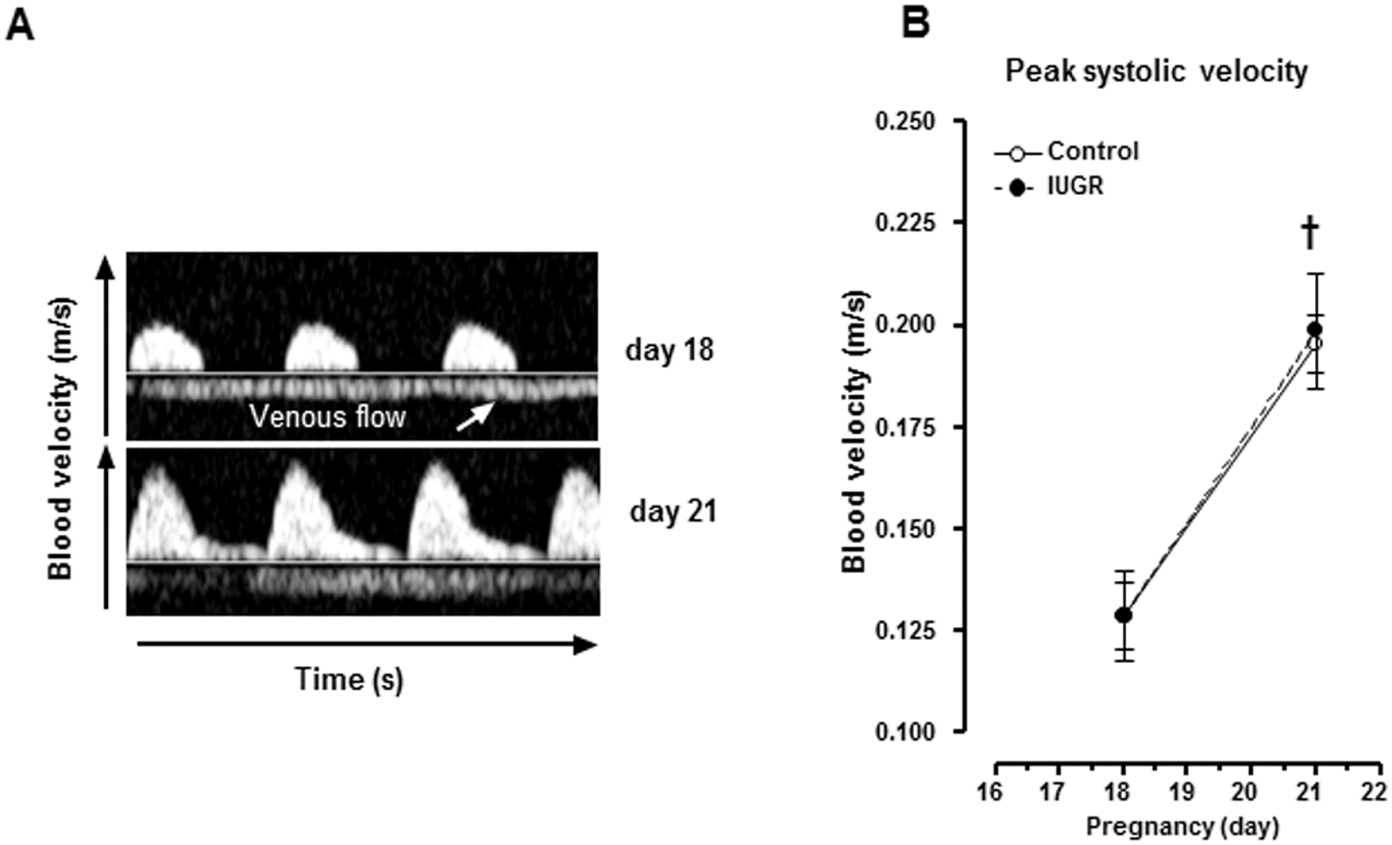
Umbilical circulation in control and IUGR animals. (A) Doppler velocity waveforms recorded from the umbilical cord of a control fetus on day 18 and 21 of gestation. (B) Peak systolic velocity in umbilical cord. Results are expressed as means ± SEM from 8 fetuses/group. † P<0.05, two-way ANOVA, effect of gestational day.

Umbilical cord blood flow was visualised in colour mode on days 14 (data not shown), 18 and 21 (Fig 2A) in Doppler mode. These waveforms are characterised by a positive arterial blood velocity and a negative venous blood velocity within the Doppler sample volume. At day 21 of pregnancy, a positive diastolic flow waveform was detected (Fig 2A). Umbilical artery peak systolic blood velocity increased during pregnancy in both groups of rats (Fig 2B; P<0.05, two-way ANOVA). Similarly, fetal heart rate also augmented as pregnancy progressed (P<0.05, two-way ANOVA) without any difference between the two groups (data not shown). These results thus suggest that umbilical circulation is not affected by the diet.

### Myogenic responses of the uterine radial artery from control and IUGR pregnant rats

**Fig 3:**
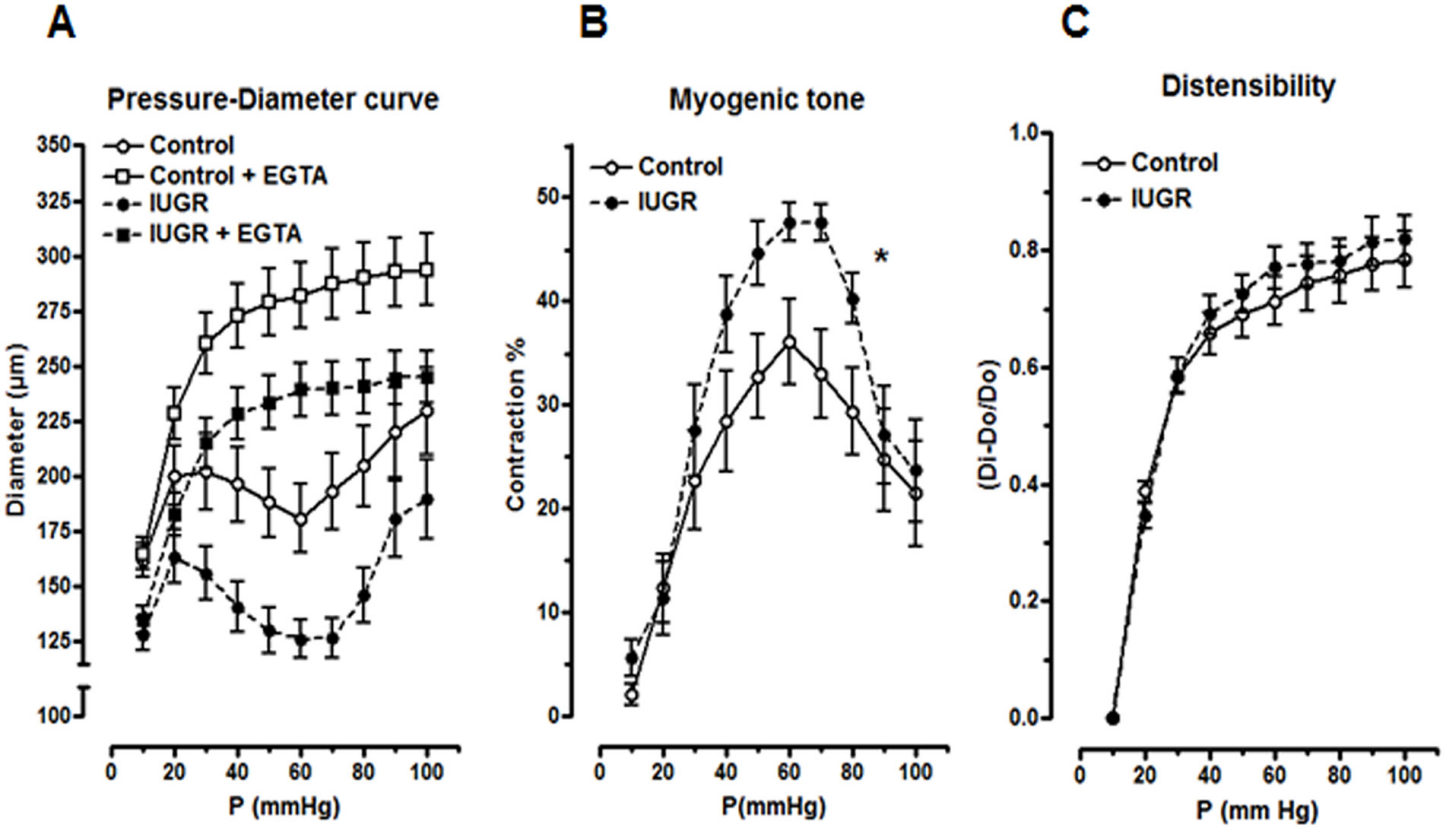
Myogenic responses of radial uterine artery supplying a placenta from control and IUGR 22-day pregnant rats. (A) pressure-diameter relationship, (B) myogenic tone and (C) distensibility. Results are expressed as means ± SEM from 18 blood vessel segments from 9 rats/group. * P<0.05, two-way ANOVA vs. control pregnant rat. Di: internal diameter at a given pressure. Do: original diameter measured at 10mmHg.

Using Doppler ultrasound, an increased blood flow resistance index in the main uterine artery in the IUGR group was observed. To identify arterial dysfunction that could explain this increase, mechanical and myogenic properties of radial arteries were examined by pressure myography. As shown in Fig 3A, in presence of calcium (1.8mM CaCl_2_), the diameter of uterine radial arteries supplying the placenta varied with intraluminal pressure in three phases, analogous to the model proposed by Osol et al [27]. First, there was passive arterial distension until 20 mmHg, after which blood vessel segments contracted (myogenic response) with a decreased diameter to 193 ± 17 at 60 mmHg in control pregnant rats, followed by what resembled “forced dilation” in the third phase. Radial arteries from the IUGR group followed a similar pattern; however, the uterine radial arteries constricted to a smaller minimum diameter of 127 ± 9 μm at 60 mmHg. The pressure-diameter curves in presence of EGTA showed that the myogenic response was dependent on extracellular calcium influx since vascular segments dilated passively. Passive diameter of the uterine radial artery from IUGR pregnant rats was significantly smaller (P<0.05, two-way ANOVA) compared to control rats indicating that pregnancy remodeling of this artery was blunted. Conversely, myogenic tone (calculated from the pressure-diameter curves) was increased in radial artery from IUGR rats compared to controls (Fig 3B; P <0.05, two-way ANOVA), especially in the 40–80 mmHg pressure range. Distensibility did not differ between the two groups (Fig 3C). Altogether, these results suggest that IUGR induction in the current model brings some alteration in uterine radial artery remodeling with increased myogenic behaviour.

### Placental hypoxia markers and apoptosis

**Fig 4:**
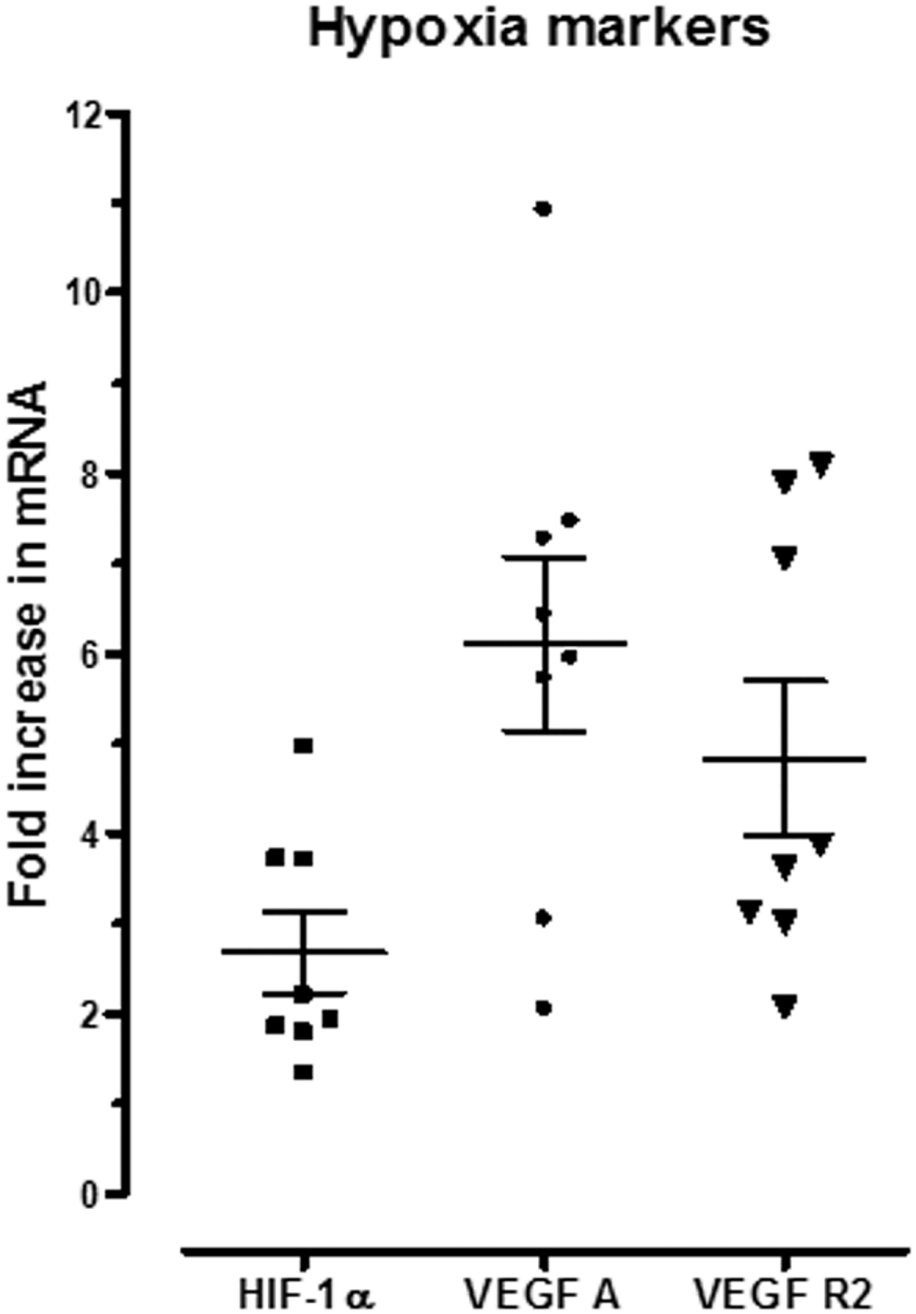
Expression of hypoxia markers in placenta from IUGR 22-day pregnant rats compared to their controls. Relative placental mRNA expression of HIF-1α, VEGFA and VEGFR2 in placenta from term IUGR placenta. Each point represents one placenta. Ct average of each duplicate was calculated for each gene and 18S and the ΔCT (CTgene—CT18S) was determined. The control placental tissue sample was chosen as a reference sample and set as 100% of gene quantity. The mRNA abundance of the placenta was calculated with the formula 2-ΔΔCT. Results are expressed as means ± SEM. (n = 8 rats/genes). * P<0.05, one sample Student’s t test.

Impaired uterine blood velocity and increased myogenic response in the IUGR model could lead to placental hypoxia and/or apoptosis. Relative mRNA expression levels of placenta hypoxia markers were measured. As shown in Fig 4, HIF-1α, VEGFA and VEGFR2 were increased in IUGR placenta compared to control ones by 3-, 6- and 5-fold, respectively (P<0.05). VEGFA protein quantity, determined by ELISA, was also augmented in IUGR placenta (13.0 ± 0.4 vs 10.9 ± 0.5 pg/mL, p<0.05). These results suggest presence of hypoxia in the term-IUGR placenta.

**Fig 5:**
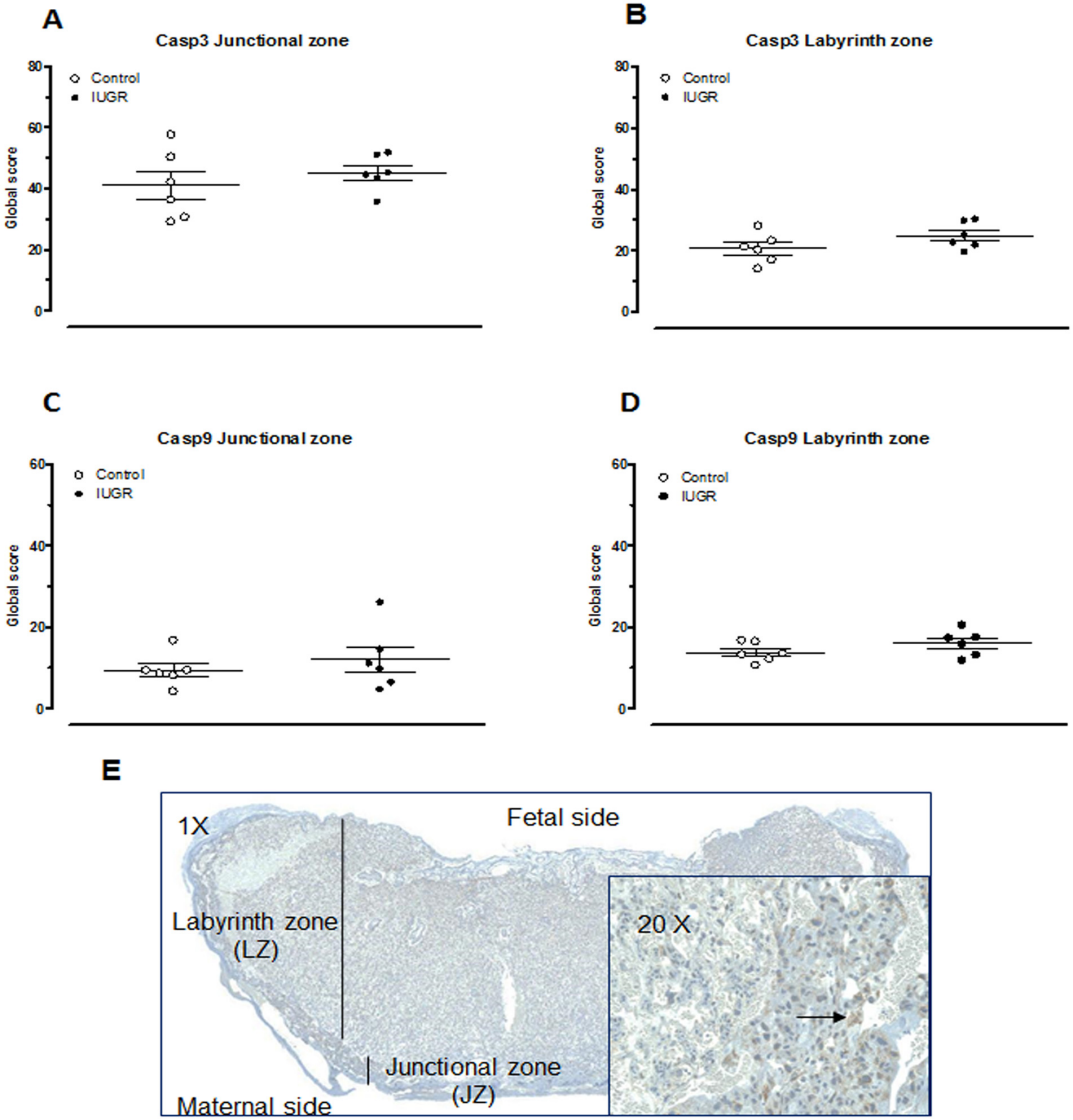
Immunostaining of caspase-3 (A, B) and -9 (C, D) in placenta from control and IUGR 22-day pregnant rats. Quantification of caspase 3 (cleaved) and 9 expression in the junctional (A, C) and labyrinth (B, D) zones. Each point represents one placenta. Global Score = (MI x Area)LOW + (MI x Area)MOD + (MI x Area)STRONG / Total Area of ROI (see Methods). (n = 6 rats/group). Representative immunostaining of cleaved caspase-3 from placenta of control rats (E). Inset: Arrow indicates brown positive stains.

In an attempt to characterise placenta apoptosis, expression level for caspase 3 (cleaved) and 9 were determined by IHC in placental junctional and labyrinth zone. There was no difference in both zones between IUGR and control placentas (Fig 5).

### Placental histology

**Fig 6:**
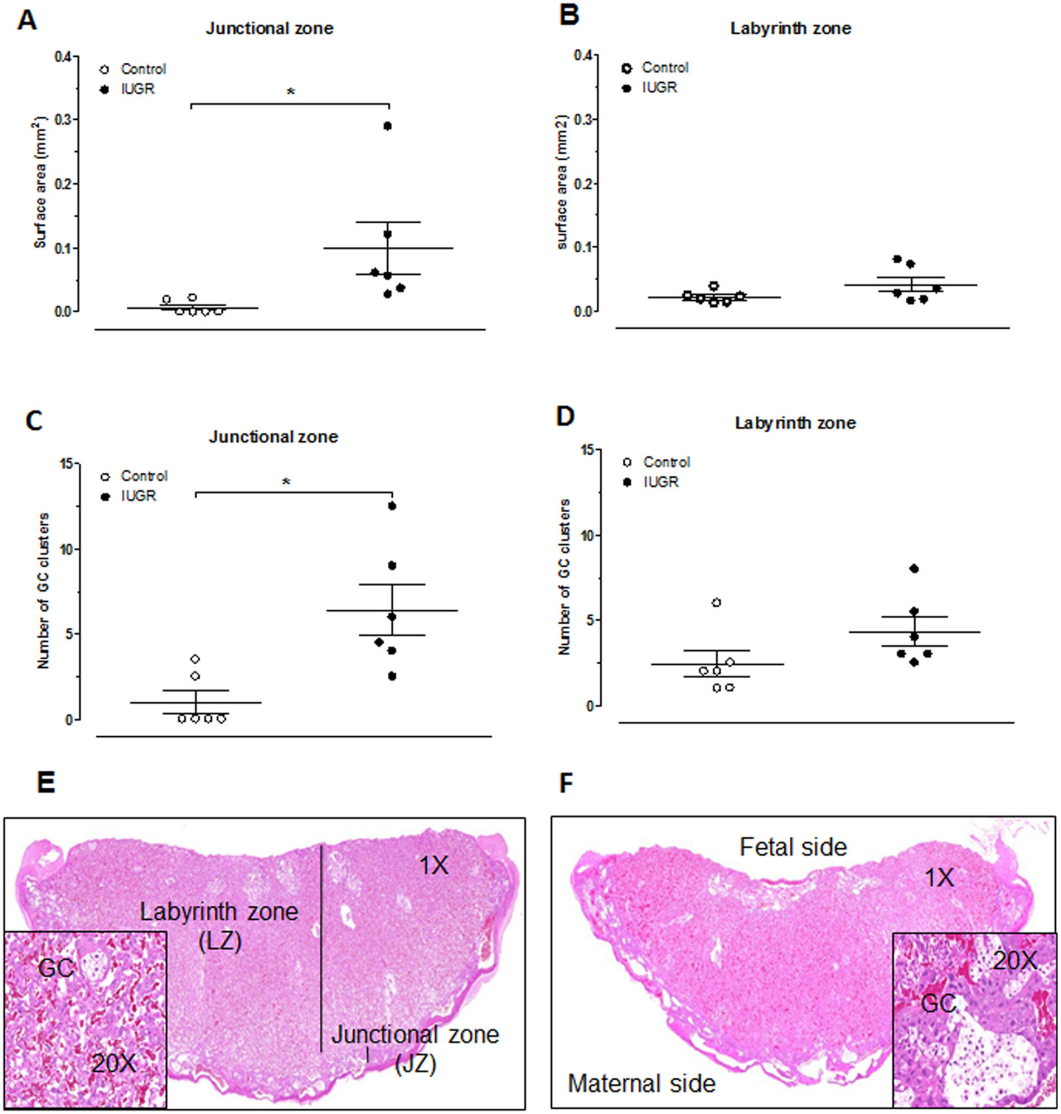
Presence of glycogen cell clusters (GC) in placenta from control and IUGR 22-day pregnant rats. Surface area of GC clusters in junctional (A) and labyrinth (B) zones. Number of GC clusters in junctional (C) and labyrinth (D) zones. Each point represents one placenta. Results are expressed as means ± SEM from 6 rats/group. *P<0.05 vs. control pregnant rat, Student’s t test. Representative placenta of control (E) and IUGR (F) rat (HE stain).

To better characterise the effect of reduced plasma volume expansion on placenta development, histological analyses were done. No gross morphological abnormalities in term-IUGR placentas were observed. However, surface area occupied by glycogen cells (GC) and number of GC clusters were greater in junctional zone of IUGR placentas compared to the control ones (p<0.05, Fig 6A and 6C, respectively). In the labyrinth zone, no differences of GC clusters (surface area and number) were observed between the two groups (Fig 6B and 6D).

## Discussion

Reduced plasma volume expansion during human pregnancy is associated with IUGR [28]. This study was aimed at characterising uteroplacental circulation and placental phenotype in an IUGR rat model associated with decreased maternal volume. Results herein reveal 1) an impaired blood flow in the main uterine artery with an increased resistance index, but without alteration in umbilical artery blood flow; 2) reduced diameter and increased myogenic response of the radial uterine artery; 3) increased placental expression of hypoxia markers; 4) augmented glycogen cells in junctional zone in the IUGR model.

### Uteroplacental circulation in IUGR pregnant rats

There was no difference in umbilical artery systolic velocity between control and IUGR fetuses, thus suggesting that impaired fetal blood flow was not a cause of growth restriction. However, placental weight is reduced in this IUGR model [24]. It can be speculated that the exchange surface area between mother and fetuses is likely reduced. Furthermore, uterine blood flow was shown to be inadequate for optimal fetal growth in this rat model.

In control rats, the increased systolic and diastolic blood velocities in the main uterine artery observed over the last week of gestation were accompanied by a simultaneous reduction in resistance index. This suggests the establishment of a low resistance upstream blood flow therefore increasing placental perfusion. This is in accordance with previous works in rat [29, 30], mouse [31], rabbit [32] and ewes [33]. During the last week of rat pregnancy, both fetal mass and placental weight increase exponentially [8] and are accompanied by an enhancement of blood flow to the uterine tissue and placenta. At day 15 of gestation, less than 10% of uterine blood flow is directed to the placenta whereas, at near-term, this value reaches 90% [8, 29]. This increased uterine blood flow is essential in order to adequately respond to the needs of the growing fetuses. In our IUGR model, at day 21 of pregnancy, both systolic and diastolic blood velocities in the main uterine artery were reduced compared to their controls. No decrease in resistance index was noted during the last week of gestation confirming a diminished placental perfusion that may be induced by a potential defect in the remodeling of the uterine radial artery as illustrated by the smaller diameter in passive pressure-diameter curves. Indeed, to ensure an adequate perfusion, uterine vasculature undergoes extensive remodeling involving various mechanisms such as trophoblastic invasion, shear stress, intravascular pressure, humoral factors, etc. (for review, see [34]). The observed increase in blood flow could in part be responsible for the remodeling as shown by Pourageaud [35]. Indeed, the author demonstrated that ligation of second-order side branches of the superior mesenteric artery reduced blood flow in the vessels feeding into the ligated trees while elevating blood flow in the non-ligated mesenteric artery side branches. This high blood flow resulted in outward hypertrophic remodeling involving hyperplasia. Furthermore, contrary to normal pregnancy, plasma volume expansion is blunted in the IUGR model [24], thus partially preventing remodeling of uterine radial artery. Decreased trophoblast invasion could also contribute to the alteration in the remodeling. Indeed, GC islands are increased in the junctional zone of the IUGR term-placenta. This could suggest decreased trophoblast invasion that lead to alteration in vascular remodeling. Multiple signaling pathways were identified in trophoblast primary culture and cell lines to explained trophoblast invasion and migration [36]. However, the physiological phenomenon remains to be elucidated.

Vascular remodeling in pregnancy is accompanied by an increase in vascular contractility to vasoconstrictor agents such as angiotensin II and phenylephrine [7, 9, 11] or in response to pressure (myogenic tone) [9, 37]. This latter phenomenon is thought to maintain perfusion pressure at an adequate level for optimal nutrient exchange in the placental labyrinth [37]. The radial artery myogenic tone observed in control pregnant rats herein is comparable to that reported by Telezhkin and coll. [37]. However, in our IUGR group, myogenic tone was further increased; this phenomenon combined with the reduced diameter of the radial artery could have compromised placental perfusion. As rat and human trophoblast-directed remodeling of uterine spiral arteries exhibits striking similarities occurring in the last week of gestation in rat and in the end of the first trimester in human [38], we could suggest that remodeling of radial arteries could be impaired in pregnant women having smaller plasma volume expansion and idiopathic growth restricted fetuses [28]. This is supported by a case control study in humans in which maternal and fetal hemodynamics of gestational hypertensive pregnancies with IUGR was improved by plasma volume expansion, nitric oxide donors and antihypertensive drug therapy [39]. These findings thus illustrate the importance of plasma volume expansion in normal pregnancy. Of note, Leandro and coll. [40] conversely reported that growth restriction induced by low sodium diet in pregnant rats was not a consequence of reduced uterine blood flow. Certain differences may be raised to explain the discrepancy between their data and the findings herein, including amount of sodium in the diet, diet duration, time of pregnancy and limited animal number in their blood flow study by radioactive microsphere.

In the present model, low sodium diet given during the last third of pregnancy in the rat induced insufficient expansive remodeling with augmented vascular tone of the radial artery supplying the placentas. This finding explains the augmented resistance index observed in the main uterine artery during the Doppler ultrasound since resistance is inversely proportional to the fourth power of the radius of an artery. This increase in resistance decreases blood flow to the placenta. Together, the above data would suggest that an inadequate uteroplacental blood flow is one of the major parameters responsible for the IUGR induced by the low sodium diet.

### Placental hypoxia markers in IUGR pregnant rats

Our results show an increase in HIF-1α, VEGF and VEGF R2 mRNA and VEGF protein expression. Hypoxia inducible factor is a key regulator of placental vascularization and invasion as well as trophoblast differentiation and thus, is essential in placental development, particularly during the first trimester of human pregnancy where the placenta develop in a hypoxic environment [41]. HIF-1α triggers the transcription of angiogenesis genes like VEGF and its receptors among others. Zamudio et al. reported increased HIF-1α mRNA and protein expression in placenta from women living at high altitude (>3100m, low oxygen pression)[20]. Furthermore, HIF-1α mRNA was increased in placenta and maternal blood of severe preterm growth restriction [42]. These are in accordance with our results. However, besides hypoxia, other stimuli such as labor [42], angiotensin II [43] and angiotensin II type 1 receptor autoantibody [44] could regulate HIF. In the present growth restriction model, activation of the renin-angiotensin-aldosterone system is increased compared to normal pregnancy [24]. Since angiotensin receptor subtype 1 is decreased in the IUGR placenta [25], hypoxia is presumably responsible for HIF-1α increase which in turn enhanced VEGF transcription and translation [45]. These results could suggest the presence of placental hypoxia in the present IUGR animal model. In IUGR human placentas, studies reported inconsistencies relative to the levels of VEGF, at times describing higher [46], unaltered [47] or lower [48] expression, although overall available data indicate that placental VEGF expression may be up-regulated in cases of IUGR in which placental hypoxia is present [49]. Furthermore, in the rat placenta, VEGF expression has been shown to increase specifically in the labyrinth zone over the last week of gestation, a change coinciding with a marked elevation of vascularisation in this zone [50]. This elevation has been proposed as an expected change aimed at enhancing the efficiency of fetomaternal transport in order to support the greater demand of the fetus during late gestation [50].

Apoptosis is necessary for normal placental development through its participation in endovascular invasion and blood vessel remodeling [51]. Increased trophoblast apoptosis was reported in placentas from IUGR human pregnancies [52, 53] and rat gestations [54, 55]. In the present IUGR model, placental expression of caspase 3 (cleaved) and 9 were not different between IUGR and control groups. Thus, our results could not confirm the presence of variations in the level of apoptosis in this model.

In summary, results obtained using the present IUGR animal model suggest that reduced maternal plasma volume expansion leads to decreased placental and fetal growth that may be mediated, in part, by decreased trophoblast invasion, alteration of uterine arteries remodeling and placental hypoxia. Longitudinal studies will be undertaken to determine the primary events that lead to this placental and fetal growth restriction.
